# *NINJ2 *SNP may affect the onset age of first-ever ischemic stroke without increasing silent cerebrovascular lesions

**DOI:** 10.1186/1756-0500-5-155

**Published:** 2012-03-20

**Authors:** Dong-Eog Kim, Sang-Mi Noh, Sang-Wuk Jeong, Min-Ho Cha

**Affiliations:** 1 Department of Neurology, Dongguk University Ilsan Hospital, Dongguk University College of Medicine, Goyang, Republic of Korea; 2 Molecular Imaging and Neurovascular Research (MINER) Laboratory, Dongguk University Ilsan Hospital, Goyang, Republic of Korea; 3 Brain Research Center, Korea Institute of Oriental Medicine, Daejeon, Republic of Korea; 4 Division of Stroke Medicine, Department of Neurology, Dongguk University Ilsan Hospital, 814 Siksa-dong, Goyang 410-773, Republic of Korea

## Abstract

**Background:**

To investigate if single nucleotide polymorphisms on chromosome 12p13 and within 11 kb of the gene *NINJ2 *would be associated with earlier-onset (vs. late-onset) first-ever ischemic stroke and increase silent cerebrovascular lesions prior to the manifestation of the stroke.

**Methods:**

We prospectively enrolled 164 patients (67.6 ± 12.9 years, 92 men) admitted with first-ever ischemic strokes. All patients underwent genotyping of rs11833579 and rs12425791 as well as systemic investigations including magnetic resonance (MR) imaging and other vascular workup. Stroke-related MR lesions were registered on a brain-template-set using a custom-built software package 'Image_QNA': high-signal-intensity ischemic lesions on diffusion, T2-weighted, or fluid attenuation inversion recovery (FLAIR) MR images, and low signal intensity hemorrhagic lesions on gradient-echo MR images.

**Results:**

The rs11833579 A/A or G/A genotype was independently associated with the first-ever ischemic stroke before the age 59 vs. 59 or over, after adjusting for cardiovascular risk factors and prior medication of antiplatelet or anticoagulant drugs, increasing the risk by about 2.5 fold. In the quantitative MR lesion maps from age-sex matched subgroups (n = 124 or 126), there was no difference between the patients with the rs11833579 A/A or G/A genotype and those with the G/G genotype. Unexpectedly, the extent of leukoaraiosis on FLAIR-MR images tended to be smaller in the corona radiata and centrum semiovale of the patients with the rs12425791 A/A or G/A genotype than in those with the G/G genotype (*P *= 0.052). Neither the rs11833579 nor the rs12425791 genotype significantly affected initial stroke severity; however the latter was associated with relatively low modified Rankin scale scores at 1 year after stroke.

**Conclusions:**

The rs11833579 A/A or G/A genotype may bring forward the onset age of first-ever ischemic stroke without increasing silent cerebrovascular lesions prior to the stroke. Further studies are required to confirm our preliminary findings.

## Background

Ischemic Stroke is a leading cause of death and disability worldwide. The South London Stroke Register, a population based study of stroke incidence and outcome, showed that only a third of survivors had returned to paid work at 1 year [1]. According to the results to estimate projected costs of ischemic stroke in the United States, loss of earnings was expected to be the highest contributor to the future economic burden of stroke, constituting nearly one-third of the total costs [2]. This burden is multiplied if the stroke occurs earlier in life. The prediction of the stroke onset age would contribute to better prevention of stroke by allowing more individualized strategies such as control of modifiable vascular risk factors in a more precise and intense manner. However, there is limited information on factors influencing the onset age of first-ever ischemic stroke.

Recently, intergenic single-nucleotide polymorphisms (rs11833579 or rs12425791 SNP) on chromosome 12p13 and within 11 kb of the gene *NINJ2 *that encodes ninjurin2, an adhesion molecule expressed in glia and shows increased expression after nerve injury [3,4], were reported to be associated with total, ischemic, and atherothrombotic stroke in white persons [5]. Thereafter, it was followed by controversial results in different populations [6-9]. In the Japanese population, rs12425791 was significantly associated with atherothrombotic stroke: both small artery occlusion and large artery atherosclerosis [6]. However, many studies including a Chinese Han study failed to replicate the association of either rs11833579 or rs12425791with ischemic stroke [7-9]. In the field of stroke, including the *NINJ2*-related studies, failure to find replicated SNP effects across population-based studies has significantly limited the utility of genetic association results [10]. Stroke cannot be regarded as a single disease entity but as a heterogeneous syndrome, with more than 150 known causes [11], under multiple genetic and clinical influences; and the effect size of a single SNP could be rather small in predicting stroke risk in the general population. Moreover, roles for a genetic factor in the general population vs. stroke population may not be identical. In addition, the influence of genetic factors might be more pronounced in the younger, before classic cardiovascular risk factors exert their full influence on the disease, although the genetic and other risk factors could work in concert and produce stroke at a later age [12]. Taken together, these led us to reason that clinical implications for the *NINJ2 *SNP, including a potential effect on the onset age of the first-ever ischemic stroke, need to be further elucidated 'within a carefully characterized stroke population' in a detailed manner that includes quantitative analyses of magnetic resonance (MR) images, by which many of diagnostic and therapeutic decisions are today guided in clinic. Until now, the impact of the *NINJ2 *SNP on stroke-related lesions on multi-sequence MR images has not been studied.

In the present study, we tried to see if the *NINJ2 *SNP (A allele) would be associated with earlier-onset (vs. late-onset) first-ever ischemic stroke. This study, conducted in a hospital-based stroke cohort, could be underpowered to detect small effects of the single SNP on the development of the clinical endpoint [10,13]. Thus, in an effort to corroborate the associations, we used quantitative data on stroke-related MR lesions [14] as endophenotypes—subclinical phenotypes that are heritable traits, revealing the actions of genes predisposing an individual to develop conditions known to increase the risk of future disease, often having a larger genetic component than clinical disease endpoints, and manifesting years before clinical diagnostic criteria for the disease are met [13]. Considering that unrecognized or covert brain infarcts and white matter lesions are linked to increased risk for stroke [15], we investigated if the *NINJ2 *A/A or G/A genotype increased silent cerebrovascular lesions such as leukoaraiosis, asymptomatic lacunes, or microbleeds prior to the manifestation of first strokes. Moreover, we studied if the *NINJ2 *genotypes affected post-stroke functional outcome at 1 year.

## Methods

This study was approved by the institutional review board of Dongguk University Ilsan Hospital (DUIH).

### Patients

Out of 586 consecutive first-ever ischemic stroke patients admitted to our hospital, a community-based academic hospital at a suburban city (Goyang) in Seoul Metropolitan Area, from 2008 February to October 2010, we prospectively enrolled 164 patients (67.6 ± 12.9 years, 92 men) who gave written informed consent to "DUIH stroke-genetic-study project". They did not have a history of hemorrhagic stroke, either. All patients underwent systemic investigations including assessment of ischemic cerebral events, medication history, MR imaging with MR angiography, carotid duplex ultrasonography, transthoracic echocardiography, 24 h Holter monitoring, and other routine admission laboratory tests. In selected patients, transesophageal echocardiography or transcranial Doppler bubble study was also performed. Under a standardized protocol, we prospectively collected demographic data, prior medication history, and the presence of vascular risk factors including hypertension, diabetes mellitus, dyslipidemia, heart disease, and smoking.

### Risk factor variables and stroke classification

Hypertension was considered to be present if a subject had one of the following conditions: repeated blood pressure readings above 140/90 mmHg at 1 ~ 2 weeks after stroke onset, a history of hypertension, or use of antihypertensives. Diabetes mellitus was defined as glycated hemoglobin (HbA1C) ≥ 6.5%, history of diabetes mellitus, or use of diabetic medication. Dyslipidemia was defined as total cholesterol ≥ 240 mg/dl, low density lipoprotein cholesterol ≥ 160 mg/dl, a history of dyslipidemia, or use of lipid lowering agents. Heart disease as potential source of cerebral embolism was considered to be present if a subject had left atrial or ventricular thrombus, atrial fibrillation or sick sinus syndrome, recent myocardial infarction within 1 month, rheumatoid mitral or aortic valve disease, bioprosthetic or mechanical heart valves, chronic myocardial infarction with low ejection fraction less than 28%, symptomatic congestive heart failure with ejection fraction less than 30%, dilated cardiomyopathy, aneurysms or akinetic segments of the left ventricular wall, or nonbacterial thrombotic endocarditis. Smokers included current smokers or those who had stopped smoking for less than 1 month. Subtypes of index stroke were determined by the consensus of three neurologists as follows; large artery atherosclerosis, small vessel occlusion, cardiac embolism, and ischemic stroke of undetermined etiology, as described in Trial of Org 10172 in Acute Stroke Treatment (TOAST) [16].

### Genotyping

The genomic DNA of each subject was extracted from peripheral blood mononuclear cells using a Type G Genomic DNA Extraction Kit (GeneAll, Seoul, Korea) according to the manufacturer's instructions. Genotyping of rs11833579 or rs12425791 was conducted using ABI BigDye^® ^Terminator v3.1 Cycle Sequencing Kits (Aplied Biosystems, Carlsbad, California); genotyping primers and representative chromatograms of the *NINJ2 *SNPs are shown (see Additional file 1: Table S1 and Additional file 2: Figure S1, respectively). Hardy-Weinberg equilibrium tests were conducted to determine whether individual variants were in equilibrium at each locus. Linkage disequilibrium (LD), Lewontin's D' (|D'|), and *r^2 ^*between pairs of bi-allelic loci were measured [17].

### Brain MR lesion mapping

All patients have had their MR lesions prospectively segmented and registered on the **ch2better **brain template http://www.mccauslandcenter.sc.edu/CRNL/, for which a custom-built software package Image_QNA [14] was used. The software stores the shape and size of each lesion mapped in the standard template coordinate system, available for future analysis. The registered data includes high signal intensity ischemic lesions on diffusion, T2-weighted, or fluid attenuation inversion recovery (FLAIR) MR images, and low signal intensity hemorrhagic lesions on gradient-echo (GE) MR images. While blinded to patient identification, a trained research assistant performed the segmentation and registration under supervision of an experienced neurologist who can correct the data as needed after reviewing every registration output. The Image_QNA-based registration of MR lesions showed low inter- and intra-user variability. Groups of subjects could be visually grouped together by generating lesion accumulation maps, in which summed images are used to display the visual information of many subjects on a single image set [14,18].

### Statistical analysis

We dichotomized the age quartiles *a priori *into the first quartile (58 and below, earlier-onset group) and the three highest quartiles combined (59 and above, late-onset group), based on the minimum retirement pension age (59) in Korea. Between the groups, we compared the frequency of the *NINJ2 *SNP (GA or AA alleles) and conventional risk factors including hypertension, diabetes mellitus, dyslipidemia, heart disease, and smoking. After considering the relationship between the age/sex and stroke severity/recovery, we reasoned that age-sex matching was needed in the analysis to see the relationship between the *NINJ2 *SNP and stroke severity or recovery or quantitative MR data.

The Student's *t *test was used for comparison of normally-distributed continuous variables between groups. Chi-square test was used to compare proportions between groups. A logistic regression model was used to test statistical significance of the interaction between the *NINJ2 *SNP and conventional risk factors in predicting the earlier-onset of first-ever ischemic stroke. We also tried to adjust the results for prior medications. In age-sex matched samples, multivariate analyses were performed to determine if the *NINJ2 *SNP was an independent predictor for admission NIHSS score or 1 year mRS score. All statistical analyses were performed using a software package (SPSS 18.0, Chicago, IL). A value of *P *< 0.05 was considered statistically significant.

## Results

### Lower stroke onset ages in the patients with the rs11833579 A/A or G/A genotype than in those with the G/G genotype

Out of 586 first-ever ischemic stroke patients admitted to our hospital from 2008 February to October 2010, 164 patients who gave informed consent were enrolled. Between the enrolled patients and non-enrolled patients, there was no significant difference in age (67.5 ± 12.4 years vs. 68.0 ± 12.2 years), male gender (54.9% vs. 53.6%), and initial NIHSS score (5.0 ± 4.6 vs. 5.3 ± 5.9) (all *P *> 0.05). However, dyslipidemia was more frequent and heart disease was less frequent in the enrolled group than in the non-enrolled group. The use of statin was more frequent and the use of antihypertensive drugs was less frequent in the enrolled group than in the non-enrolled group. Stroke etiologies were statistically different between the groups. Premorbid mRS was higher and 1 year mRS was lower in the enrolled group than in the non-enrolled group (see Additional file 3: Table S2).

Minor allele frequencies (MAFs) of rs11833579 and rs12425791 *NINJ2 *SNP were 0.19 and 0.06, respectively, which were slightly lower than MAFs of other ethnicities (see Additional file 4: Table S3) registered in the International HapMap http://hapmap.ncbi.nlm.nih.gov. The two SNPs satisfied Hardy-Weinberg equilibrium (*P *> 0.05). Genotype frequencies of rs11833579 were 48.2% (G/G), 43.9%, (G/A) and 7.9% (A/A). Those of rs12425791 were 60.4% (G/G), 36.0% (G/A), and 3.7% (A/A). The LD coefficients between the two SNPs were |D'| = 0.864 and r^2 ^= 0.484 (*P *< 0.01), which suggested that these two SNPs were not linked in the Korean stroke population.

**Figure 1:**
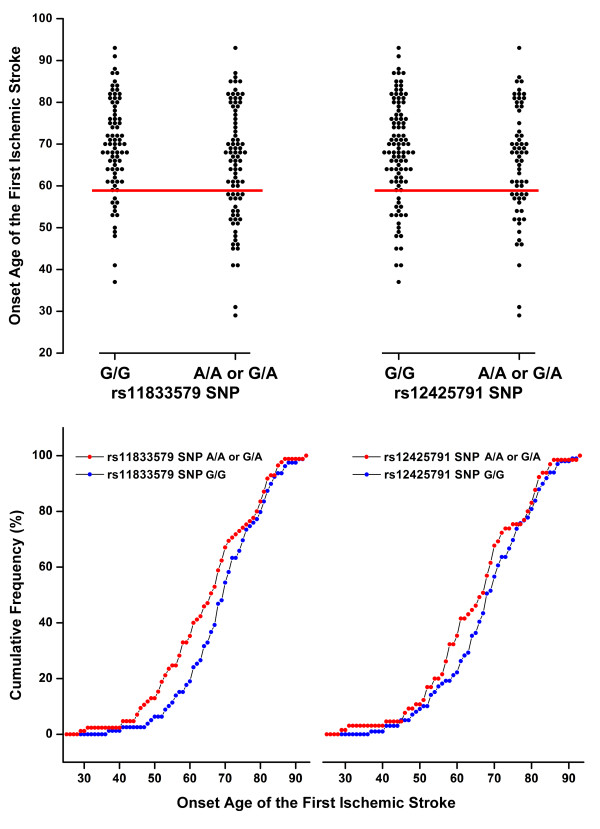
**Earlier-onset of first-ever ischemic stroke in the patients with the rs11833579 G/A or A/A SNP than in those with the G/G type**.

The ages of the patients with the A allele of rs11833579 SNP (G/A or A/A genotype; 65.2 ± 13.5 years) were lower than those without (G/G genotype; 69.4 ± 11.4 years, *P *= 0.032). The ages of the patients with the A allele of rs12425791 SNP (G/A or A/A genotype; 65.5 ± 13.3 year) were lower than those without (G/G genotype; 68.4 ± 12.2 year), which however did not reach a statistical significance (*P *= 0.150). Figure 1 shows the difference of cumulative percentage of stroke-onset-age between the rs11833579 A/A or G/A genotype group and the G/G genotype group; the gap appears to begin being narrowed around the age of 60. Around the age of 59, the cumulative frequency of first-ever stroke is about two-fold higher in the rs11833579 A/A or GA genotype group than in the G/G genotype group: approximately 40% vs. 20%. Around the age of 70, the genetic predisposition to stroke seemed to be overwhelmed by the age effect.

### More frequent rs11833579 A/A or G/A genotype in the first-ever ischemic stroke patients aged younger than 59 compared with those aged 59 or older

When the stroke onset age was dichotomized at the first quartile (= age 59), rs11833579 A/A or G/A genotype was more frequently found in the earlier-onset group than in the late-onset group (28/40 vs. 57/124, *P *= 0.008) (Table 1). Rs12425791 A/A or G/A genotype showed a similar tendency (21/40 vs. 44/124, *P *= 0.056). In addition, male sex and smoking were more frequent in the earlier-onset group than in the late-onset group. Diabetes mellitus was non-significantly more frequent and prior medication of antidiabetics was significantly more frequent in the late-onset group than in the earlier-onset group. No significant inter-group difference was observed in other vascular risk factors and stroke subtypes. The late-onset group had higher premorbid mRS scores, more severe strokes, and less favorable outcomes than the earlier-onset group had.

**Table 1:** Univariable analysis: factors associated with earlier-onset (< 59 years) vs.late-onset (≥ 59) first-ever ischemic stroke

	Earlier-onset (n = 40)	Late-onset (n = 124)	P*
Age	50.2 ± 7.1	72.8 ± 8.4	< 0.001

Sex (male)	34 (85.0%)	56 (45.2%)	< 0.001

Rs12425791 genotype (GA or AA)	21 (52.5%)	44 (35.5%)	0.056

Rs11833579 genotype (GA or AA)	28 (70.0%)	57 (46.0%)	0.008

Hypertension	25 (62.5%)	91 (73.4%)	0.188

Diabetes mellitus	15 (37.5%)	53 (42.7%)	0.558

Dyslipidemia	31 (77.5%)	79 (63.7%)	0.107

Heart disease	1 (2.5%)	10 (8.1%)	0.298^†^

Smoking	31 (77.5%)	55 (44.4%)	< 0.001

Current smoking	30 (75.0%)	50 (41.3%)	< 0.001

Prior medication			

Antihypertensives	11 (27.5%)	52 (41.9%)	0.103

Antidiabetics	9 (22.5%)	38 (30.6%)	< 0.001

Statins	10 (25.0%)	37 (29.8%)	0.556

Antiplatelet	8 (20.0%)	23 (18.5%)	0.838

Warfarin	0 (0.00%)	1 (0.81%)	0.572

Ischemic stroke subtype^‡^			0.401^†^

Large artery atherosclerosis	11 (27.5%)	36 (29.0%)	

Small vessel occlusion	22 (55.0%)	62 (50.0%)	

Cardioembolism	2 (5.0%)	4 (3.2%)	

Other determined etiology	1 (2.5%)	0 (0%)	

Undetermined etiology	4 (10.0%)	22 (17.7%)	

Premorbid mRS	0.6 ± 1.2	1.4 ± 1.8	0.001

Admission NIHSS	3.1 ± 2.4	5.5 ± 5.9	< 0.001

Discharge NIHSS	1.7 ± 2.3	3.5 ± 4.6	0.002

NIHSS (Admission - Discharge)	1.4 ± 2.8	2.0 ± 4.9	0.421

Discharge mRS	1.4 ± 1.3	2.4 ± 1.7	< 0.001

1 year mRS (n = 39 & 117)	0.5 ± 0.8	2.0 ± 1.7	< 0.001

Values are number (percentage) or mean ± standard deviation.

*P for Student's *t *test or Chi-square test.

^†^Fisher's exact test.

^‡^TOAST classification

mRS and NIHSS denote modified Rankin scale and NIH stroke scale, respectively.

### An independent association of rs11833579 A/A or G/A genotype and male sex with the first-ever ischemic stroke before the age 59 vs. age 59 or over

To adjust for conventional stroke risk factors a multivariable analysis was performed (Table 2), which showed that rs11833579 A/A or G/A genotype and male sex were independently associated with the first-ever ischemic stroke before the age 59 vs. age 59 or over. In addition, dyslipidemia was associated with the earlier-onset stroke, which was marginally significant. When additionally adjusted for prior medication, either antiplatelet or warfarin, in another regression analysis, rs11833579 A/A or G/A genotype and male sex were again positively associated with the earlier-onset of first-ever ischemic stroke (see Additional file 5: Table S4).

**Table 2:** Multivariable analysis: factors associated with earlier-onset (< 59 years) vs.late-onset (≥ 59) first-ever ischemic stroke

Variable	Odds ratio (95% C.I.) for the earlier-onset first stroke	P
Rs11833579 genotype (GA or AA)	2.46 (1.06-5.69)	0.036

Male sex	5.73 (1.52-21.61)	0.010

Hypertension	0.50 (0.20-1.23)	0.129

Diabetes mellitus	0.95(0.40-2.24)	0.904

Dyslipidemia	2.51(0.98-6.44)	0.057

Heart disease	0.29(0.03-2.78)	0.283

Smoking	1.34 (0.40-4.49)	0.636

Hosmer-Lemeshow goodness-of-fit test showed *χ*^2 ^= 11.43 and *P *= 0.18, demonstrating a good fitness of the model. *C.I*. confidence interval.

### No significant effect of rs11833579 or rs12425791 SNP on vascular risk factors, stroke etiologies, and initial stroke severity

By matching age and sex while blinded to patient identification, we selected 126 subjects with (n = 63, 68.3 ± 11.7 years, 31 men) or without (n = 63, 68.3 ± 11.0 years, 32 men) rs11833579 A/A or G/A genotype (see Additional file 6: Table S5). There was no intergroup difference in the vascular risk factors and distribution of ischemic stroke subtypes. Statin medication was more frequent in the rs11833579 A/A or G/A genotype group than in the G/G genotype group with borderline significance (*P *= 0.052). Admission NIHSS scores were non-significantly (*P *= 0.103) lower in the former than in the latter. However, 'admission NIHSS - discharge NIHSS' scores did not show statistical differences between the groups (*P *= 0.251). At 1 year follow up, mRS scores did not differ between the groups, either (*P *= 0. 649). Moreover, in a conditional logistic regression analysis for the matched subgroup data, no parameters were significantly associated with the rs11833579 A/A or G/A genotype vs. G/G genotype (see Additional file 7: Table S6). Another multivariable linear regression analysis to adjust for statin medication and premorbid mRS showed that not the rs11833579 A/A or G/A genotype but premorbid mRS was significantly associated with admission NIHSS scores (see Additional file 8: Table S7).

We also selected 124 age-sex matched subjects with (n = 62, 66.6 ± 12.0 years, 35 men) or without (n = 62, 66.6 ± 11.1 years, 34 men) the rs12425791 A/A or G/A genotype (Table 3). Between the groups, there was no difference except that 1 year mRS scores were non-significantly (*P *= 0.143) lower in the rs12425791 A/A or G/A genotype group than in the G/G genotype group. In a multivariable analysis after adjusting for premorbid mRS and admission NIHSS that turned out to be a significant predictor (*P *< 0.001) for the mRS score at 1 year, rs12425791 GA or AA genotype was a significant negative predictor of mRS score at 1 year (Model I of Table 4).

**Table 3:** Clinical profiles of age-sex matched patients with the rs12425791 A/A or G/A genotype vs.GG genotype

	Rs12425791 genotype		P*
		
	(A/A or G/A) (n = 62)	(GG) (n = 62)	
Age	68.3 ± 11.7	68.3 ± 11.0	0.994

Sex (male)	35 (56.5%)	34 (54.8%)	0.857

Hypertension	42 (67.7%)	46 (74.2%)	0.429

Diabetes mellitus	24 (38.7%)	24 (38.7%)	1.000

Dyslipidemia	41 (66.1%)	47 (75.8%)	0.235

Heart disease	5 (8.1%)	2 (3.2%)	0.439^†^

Smoking	32 (51.6%)	33 (53.2%)	0.857

Current smoking	31 (50.0%)	29 (46.8%)	0.526

Prior medication			

Antihypertensives	21 (33.9%)	23 (37.1%)	0.707

Antidiabetics	21 (33.9%)	24 (38.7%)	0.575

Statins	19 (30.6%)	19 (30.6%)	1.000

Antiplatelets	12 (19.4%)	12 (19.4%)	1.000

Warfarin	0 (0.0%)	1 (1.6%)	0.496^†^

Ischemic stroke subtype^‡^			1.000^†^

Large artery atherosclerosis	16 (25.8%)	17 (27.4%)	

Small vessel occlusion	35 (56.5%)	35 (56.5%)	

Cardioembolism	1 (1.6%)	2 (3.2%)	

Other determined etiology	1 (1.6%)	0 (0.0%)	

Undetermined etiology	9 (14.5%)	8 (12.9%)	

Premorbid mRS	1.2 ± 1.6	1.0 ± 1.6	0.575

Admission NIHSS	4.4 ± 3.6	4.5 ± 4.1	0.872

Discharge NIHSS	2.7 ± 3.7	2.4 ± 2.9	0.629

NIHSS (admission - discharge)	1.7 ± 3.3	2.1 ± 3.5	0.512

Discharge mRS	2.1 ± 1.6	2.0 ± 1.6	0.612

1 year mRS (n = 58 & 62)	1.2 ± 1.4	1.6 ± 1.6	0.143

Values are number (percentage) or mean ± standard deviation.

*P for Student's *t *test or Chi-square test.

^†^Fisher's exact test.

^‡^TOAST classification

mRS and NIHSS denote modified Rankin scale and NIH stroke scale, respectively.

**Table 4:** Multivariable analyses to predict modified Rankin scale (mRS) score at 1 year

Variable	Odds ratio (95% C.I.) for mRS score	P
Model I		

Rs12425791 (GA or AA)	-0.15 (-1.01-0.04)	0.036

Premorbid mRS	0.09 (-0.07-0.24)	0.262

Admission NIHSS	0.38 (0.08-0.20)	< 0.001

Model II		

Rs12425791 (GA or AA)	-0.080 (-0.618-0.150)	0.230

Premorbid mRS	0.016 (-0.122-0.152)	0.825

Admission NIHSS	0.323 (0.068-0.171)	< 0.001

Age	0.369 (0.030-0.067)	< 0.001

Pre-stroke leukoraiosis burden (per 100 pixels)	0.128 (0.000-0.016)	0.068

NIHSS and C.I. denote NIH stroke scale and confidence interval, respectively.

Leukoaraiosis burden: extent of leukoaraiosis in the corona radiata and centrum semiovale on fluid attenuation inversion recovery (FLAIR) magnetic resonance images

### A trend of smaller white matter lesion extent in the patients with the rs12425791 A/A or G/A genotype than in those with the G/G genotype

**Figure 2:**
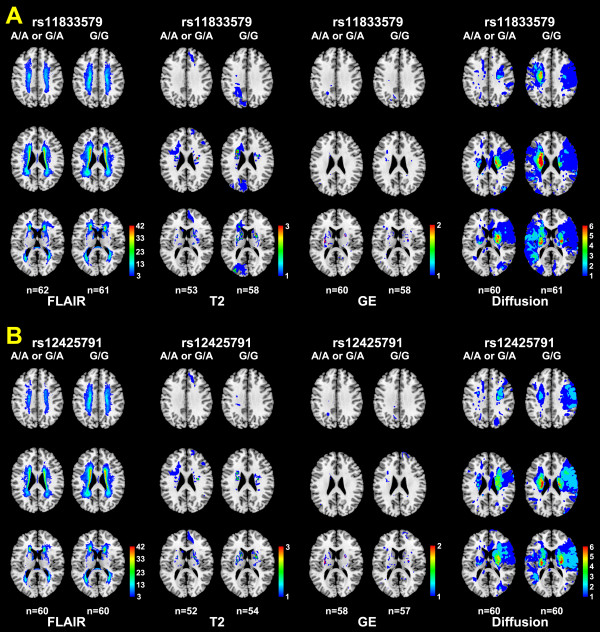
**Accumulation MR lesion maps depending on the rs11833579 (A) or rs12425791 (B) genotype**. Quantitative maps of diffusion/fluid attenuation inversion recovery (FLAIR)/T2/gradient-echo (GE) MR images show no significant difference between the age-sex matched patients with the rs11833579 A/A or G/A genotype and those with the G/G genotype (A). In the age-sex matched patients with the rs12425791 A/A or G/A genotype (B), compared with the ones with the G/G genotype, the extent of leukoaraiosis in the centrum semiovale (first row) and corona radiata (second row) on FLAIR MRIs appears to be smaller. The pseudocolor bars indicate numbers of subjects with lesions overlapping a specific pixel.

In the quantitative mapping of stroke-related lesions on diffusion/FLAIR/T2/GE MR images, there was no difference between the age-sex matched patients with the rs11833579 A/A or G/A genotype and those with the G/G genotype (Figure 2A). Likewise, there was no difference in the MR images of the age-sex matched patients with the rs12425791 A/A or G/A genotype vs. those with the G/G genotype, except that the extent of leukoaraiosis in the corona radiata and centrum semiovale tended to be smaller in the former (1861 ± 2437 pixels) than in the latter (1136 ± 1528 pixels, *P *= 0.052) (Figure 2B).

When the extent of leukoaraiosis and onset age of stroke, two parameters that could have potential links with the *NINJ2 *SNP or post-stroke prognosis, were additionally entered into the multivariable analysis model to predict mRS at 1 year (Model I of Table 4), the SNP lost significance whereas the age was a statistically significant predictor and the leukoaraiosis burden was a marginally significant predictor for the follow-up mRS (Model II of table 4).

## Discussion and Conclusions

In the present study, we found that the rs11833579 A/A or G/A *NINJ2 *genotype was an independent risk factor for the first-ever ischemic stroke before the age 59 vs. age 59 or over, increasing the relative risk by about 2.5 fold. First strokes occurred before the age 59 in 70% of the patients with the A/A or G/A genotype, but only in about 46% of the patients with the G/G genotype. Male sex was another risk factor for the earlier-onset of first stroke, which is in line with a previous report [19-22]. In the quantitative mapping of stroke-related lesions on multi-sequence MR images from age-sex matched subjects, there was no difference between the patients with the rs11833579 A/A or G/A genotype and those with the G/G genotype. Both the rs11833579 and rs12425791 genotypes did not significantly affect initial stroke severity or subsequent recovery from the stroke.

Meschia et al. reported that family history of stroke before the age of 65 years was a stronger risk factor at age 65 years or earlier, which indicates that an inherited component of stroke risk may attenuate with age [23-25]. Thus, once risk factor genes are identified, it would be informative to assess relative risk for stroke as a function of age [26,27]. We found that around the age sixty the genetic predisposition to stroke by the rs11833579 A/A or G/A genotype started being attenuated by the age effect. After adjusting for conventional risk factors and prior medication of antiplatelet or anticoagulant drugs, the association between the rs11833579 A/A or G/A genotype and the earlier-onset of first ischemic stroke remained significant. Between the age-sex matched subgroups with vs. without the rs11833579 A/A or G/A genotype, there was no difference in the extent and distribution of silent cerebrovascular lesions as well as of acute ischemic infarction. In addition, there was no intergroup difference in the vascular risk factors and distribution of ischemic stroke subtypes. These may explain the results that initial stroke severity or subsequent recovery from the stroke did not differ between the groups.

The Framingham Heart Study showed that the presence of severe leukoaraiosis at baseline more than doubles the future risk of stroke [28]. Many studies showed that leukoaraiosis volume, influenced by genetic variation in several underlying biological processes, was highly heritable [29-31]. In our study, the burden of leukoaraiosis did not differ between the patients with the rs11833579 A/A or G/A genotype and those with the G/G genotype. The mean onset age of first-ever ischemic stroke was significantly but modestly (about 4 years) younger in the patients with the A allele than in those without. These suggest that the genetic factor might work in concert with other classic risk factors [12,32] that do not affect leukoaraiosis, or induce stroke before the conventional vascular risk factors exert their full influence on the leukoaraiosis. It should be understood in a similar vein that *NINJ2 *SNPs were not associated with an increased risk of the other covet cerebrovascular lesions—clinically silent infarcts or hemorrhage—either.

We found a trend of earlier-onset stroke in the patients with the rs12425791 A/A or G/A genotype compared with those with the G/G genotype; however, the extent of leukoaraiosis in the corona radiata and centrum semiovale tended to be smaller in the age-sex matched patients with the A/A or G/A genotype than in those with the G/G genotype. The weak association of the SNP with lower leukoaraiois burden as well as with earlier-onset stroke should be further investigated in future research. The association might be a mere coincidence, however it again suggests that aggravation of small vessel disease may not be the cause of the SNP-associated earlier-onset of first-ever ischemic stroke. The Greater Cincinnati Stroke Study [33] and a single center study from Massachusetts General Hospital [34] showed that, when compared with no or mild leukoaraiosis, severe leukoaraiosis was a strong predictor of a higher mRS score at 3 or 6 months. Thus, the rs12425791 A/A or G/A genotype-related attenuation of leukoaraisosis, along with the non-significantly earlier onset of the first stroke, might have contributed to relatively low post-stroke mRS scores at 1 year. This may be supported by the results of the two-step multivariable analysis (Table 4), which showed that the *NINJ2 *SNP lost significance after entering the 'extent of leukoaraiosis' and 'onset age of stroke' into the model.

There have been conflicting reports on the association between the *NINJ2 *SNP and ischemic stroke [5-8]. This may be due to heterogeneity among different ethnic groups or false positive or false negative results, possibly being related to the small effect size of the SNP in terms of predicting the stroke risk in the general population. The present study conducted within Korean stroke patients, while having characterized the subjects in a detailed manner with prospective registration of not only alphanumeric data but also a comprehensive set of quantitative multi-sequence MR imaging data [14], may have selection bias; and, our findings need to be replicated in other population samples. In addition, our short-term cohort study did not investigate the duration of exposure to cardiovascular risk factors, quality of control of the risk factors, and environmental factors that could influence the onset age of first stroke. Despite the limitations, as a hypothesis-generating research, our study to provide with both genetic and quantitative-MR data as well as post-stroke functional outcome data merits further confirmatory investigation with a larger sample size.

To summarize, the rs11833579 A/A or G/A *NINJ2 *genotype may bring forward the onset age of first-ever ischemic stroke without affecting asymptomatic cerebrovascular lesion formation prior to the stroke and neurological and radiological severity of the stroke, and functional recovery after the stroke. The rs12425791 A/A or G/A genotype was associated with relatively low post-stroke mRS scores at 1 year.

## Competing interests

The authors declare that they have no competing interests.

## Authors' contributions

DEK conceived and designed the study. He recruited the patients and interpreted the study data, and wrote a draft. SMN and SWJ recruited the patients and participated in the intellectual discussion of the results and critical revision of the manuscript. They also participated in study design and coordination. MHC interpreted the data and participated in the intellectual discussion of the results and critical revision of the manuscript. All authors read and approved the final manuscript. Each author has participated sufficiently in the work to take public responsibility for appropriate portions of the content. All authors read and approved the final manuscript.

## Supplementary Material

Additional file 1 **Table S1**. Genotyping primers of rs12425791 and rs11833579 *NINJ*2 single nucleotide polymorphisms (SNPs). Click here for file

Additional file 2 **Figure S1**. Representative chromatograms of *NINJ2 *single nucleotide polymorphisms genotyping. Click here for file

Additional file 3 **Table S2**. Clinical profiles of non-enrolled vs. enrolled subjects. Click here for file

Additional file 4 **Table S3**. Characteristics of *NINJ2 *SNPs with MAFs of the study population (Korean stroke subjects) vs. other ethnic populations. Click here for file

Additional file 5 **Table S4**. Multivariable analysis: factors associated with earlier-onset (< 59 years) vs. late-onset (≥ 59) first-ever ischemic stroke. Click here for file

Additional file 6 **Table S5**. Clinical profiles of age-sex matched patients with the rs11833579 A/A or G/A genotype vs. G/G genotype. Click here for file

Additional file 7 **Table S6**. Conditional logistic regression analysis to find independent factors associated with the rs11833579 A/A or G/A genotype vs. G/G genotype. Click here for file

Additional file 8 **Table S7**. Multivariable analysis to predict admission NIH Stroke Scale (NIHSS) score. Click here for file
