# Supplementary material for: NINJ2 SNP may affect the onset age of first-ever ischemic stroke without increasing silent cerebrovascular lesions
Source: BMC Res Notes. 2012 Mar 20;5:155. doi: 10.1186/1756-0500-5-155 (PMC3368733; doi:10.1186/1756-0500-5-155)
Supplement: Additional file 1 — Table S1. Genotyping primers of rs12425791 and rs11833579 NINJ2 single nucleotide polymorphisms (SNPs). [file 1756-0500-5-155-S1.PDF]

Supplementary Table 1 PCR and sequencing primer set for genotyping of rs12425791 and rs11833579 SNPs of *NINJ2* gene

SNP	Direction	PCR	Sequencing
Rs12425791	Forward	ctg ttg acc ttc agg tgc cta c	ctg ttg acc ttc agg tgc cta c (forward)
	Reverse	ctt aca gat ggg tac tca cag tt	
Rs11833579	Forward	agt gag cta tga tca tga cac tg	cct cta ttc agc cag atg tat cca (reverse)
	Reverse	cct cta ttc agc cag atg tat cca	

PCR and SNP denote polymerase chain reaction and single nucleotide polymorphism, respectively.
