# Supplementary figures and images for: NINJ2 SNP may affect the onset age of first-ever ischemic stroke without increasing silent cerebrovascular lesions
Source: BMC Res Notes. 2012 Mar 20;5:155. doi: 10.1186/1756-0500-5-155 (PMC3368733; doi:10.1186/1756-0500-5-155)

Supplementary Figure 1 Representative examples of *NINJ2* genotyping

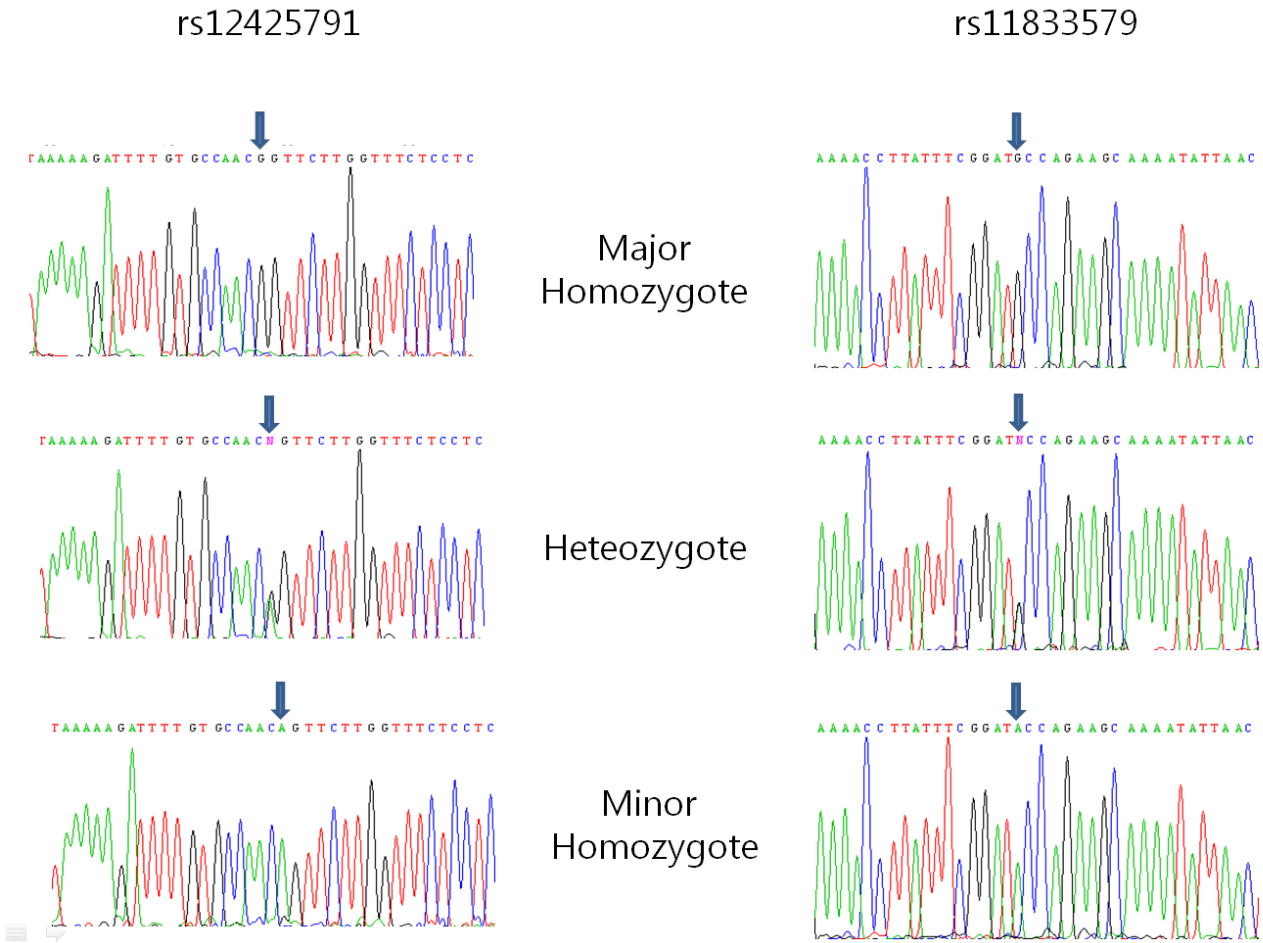

Supplement: Additional file 2 — Figure S1. Representative chromatograms of NINJ2 single nucleotide polymorphisms genotyping. [file 1756-0500-5-155-S2.PDF]
