# Supplementary material for: NINJ2 SNP may affect the onset age of first-ever ischemic stroke without increasing silent cerebrovascular lesions
Source: BMC Res Notes. 2012 Mar 20;5:155. doi: 10.1186/1756-0500-5-155 (PMC3368733; doi:10.1186/1756-0500-5-155)
Supplement: Additional file 3 — Table S2. Clinical profiles of non-enrolled vs. enrolled subjects. [file 1756-0500-5-155-S3.PDF]

Supplementary Table 2 Clinical Profiles of Non-enrolled vs. Enrolled Subjects

	Non-enrolled Subjects (n = 422)	Enrolled Subjects (n = 164)	P*
Age	68.0±12.2	67.5±12.4	0.705
Sex (male)	226 (53.6%)	90 (54.9%)	0.624
Hypertension	298 (70.6%)	115 (70.1%)	0.786
Diabetes mellitus	147 (34.8%)	68 (41.5%)	0.101
Dyslipidemia	224 (53.1%)	108 (65.9%)	0.003
Heart disease	55 (13.0%)	11 (6.7%)	0.034†
Smoking	197 (46.7%)	86 (52.4%)	0.151
Prior medication			
Antihypertensives	204 of 422 (48.3%)	63 of 164 (38.4%)	0.030
Antidiabetics	83 of 390 (21.3%)	47 of 164 (28.7%)	0.061
Statins	47 of 387 (12.1%)	47 of 164 (28.7%)	<0.001

Antiplatelet	77 of 387 (19.9%)	31 of 164 (18.9%)	0.788
Warfarin	7 of 377 (1.9%)	1 of 164 (0.6%)	0.269
Ischemic stroke subtype†			0.005
Large artery atherosclerosis	100 (23.7%)	47 (28.7%)	
Small vessel occlusion	173 (41.0%)	84 (51.2%)	
Cardioembolism	50 (11.8%)	6 (3.7%)	
Other determined etiology	5 (1.2%)	1 (0.6%)	
Undetermined etiology	94 (22.3%)	26 (15.9%)	
Premorbid mRS (n = 377 & 164)	0.6±1.2	1.3±1.7	<0.001
Admission NIHSS (n = 377 & 164)	5.3±5.9	5.0±4.6	0.471
Discharge NIHSS (n = 376 & 164)	3.8±6.1	3.0±4.2	0.086
NIHSS change (admission – discharge; n = 376 & 164)	1.6±3.7	1.9±4.5	0.492
Discharge mRS (n = 376 & 164)	2.0±1.6	2.1±1.6	0.520

1 year mRS (n = 353 & 156)	1.8±1.7	1.6±1.7	<0.001

Values are number (percentage) or mean ± standard deviation.

[*]P for Student's *t* test or Chi-square test.

[†]Trial of Org 10172 in Acute Stroke Treatment (TOAST) classification

mRS and NIHSS denote modified Rankin scale and NIH stroke scale, respectively.
