# Supplementary material for: NINJ2 SNP may affect the onset age of first-ever ischemic stroke without increasing silent cerebrovascular lesions
Source: BMC Res Notes. 2012 Mar 20;5:155. doi: 10.1186/1756-0500-5-155 (PMC3368733; doi:10.1186/1756-0500-5-155)
Supplement: Additional file 4 — Table S3. Characteristics of NINJ2 SNPs with MAFs of the study population (Korean stroke subjects) vs. other ethnic populations. [file 1756-0500-5-155-S4.PDF]

Supplementary Table 3 Characteristics of SNPs genotyped in *NINJ2* and MAFs of the study population (Korean stroke subjects) vs. other ethnic populations

SNP	Position	Chromosome locus	Locus from start codon	Change Of nucleotide	MAF_{ks}	MAF_e	MAF_h	MAF_j	MAF_y	HWE (P value)
rs12425791	Promoter	653745	-10715	G →A	0.06	0.18	0.23	0.31	0.08	0.44
rs11833579	Promoter	627705	-2443	G → A	0.19	0.23	0.30	0.34	0.22	0.90

ks: Korean stroke subjects; e: Europeans; h: Chines Hans; j: Japanese; y: Sub-Saharan Africans; HWE: Hardy-Weinberg equilibrium

SNP and MAF denote single nucleotide polymorphism and minor allele frequency, respectively.
