# Supplementary material for: NINJ2 SNP may affect the onset age of first-ever ischemic stroke without increasing silent cerebrovascular lesions
Source: BMC Res Notes. 2012 Mar 20;5:155. doi: 10.1186/1756-0500-5-155 (PMC3368733; doi:10.1186/1756-0500-5-155)
Supplement: Additional file 5 — Table S4. Multivariable analysis: factors associated with earlier-onset (< 59 years) vs. late-onset (≥ 59) first-ever ischemic stroke. [file 1756-0500-5-155-S5.PDF]

Supplementary Table 4 Multivariable Analysis: Factors Associated with Earlier-onset (< 59 years) vs. Late-onset (≥ 59) First-ever Ischemic Stroke

Variable	Odds ratio (95% C.I.) for earlier-onset first-ever ischemic stroke	P
Rs11833579 genotype (GA or AA)	2.44(1.05—5.67)	0.037
Male sex	5.85(1.53—22.37)	0.010
Hypertension	0.49(0.20—1.22)	0.125
Diabetes mellitus	0.93 (0.39—2.23)	0.871
Dyslipidemia	2.47 (0.96—6.40)	0.062
Heart disease	0.28(0.03—2.72)	0.271
Smoking	1.31(0.38—4.46)	0.669
Antiplatets or warfarin medication	1.14 (0.39—3.39)	0.811

Hosmer-Lemeshow goodness-of-fit test showed χ^2=8.25 and P = 0.41, demonstrating a good fitness of the model. C.I.: confidence interval
