# Supplementary material for: NINJ2 SNP may affect the onset age of first-ever ischemic stroke without increasing silent cerebrovascular lesions
Source: BMC Res Notes. 2012 Mar 20;5:155. doi: 10.1186/1756-0500-5-155 (PMC3368733; doi:10.1186/1756-0500-5-155)
Supplement: Additional file 6 — Table S5. Clinical profiles of age-sex matched patients with the rs11833579 A/A or G/A genotype vs. G/G genotype. [file 1756-0500-5-155-S6.PDF]

Supplementary Table 5 Clinical profiles of age-sex matched patients with the rs11833579 A/A or G/A genotype vs. G/G genotype

	Rs11833579 Genotype		P [*]
	(A/A or G/A) (n =63)	(GG) (n =63)	
Age	66.6±12.0	66.6±11.1	0.988
Sex (male)	32 (50.8%)	31 (49.2%)	0.859
Hypertension	46 (73.0%)	44 (69.8%)	0.693
Diabetes mellitus	29 (46.0%)	25 (39.7%)	0.471
Dyslipidemia	46 (73.0%)	41 (65.1%)	0.335
Heart disease	5 (7.9%)	3 (4.8%)	0.717[†]
Smoking	28 (44.4%)	31 (49.2%)	0.592
Current smoking	27 (42.9%)	29 (46.0%)	0.909
Prior medication			
Antihypertensives	25 (39.7%)	24 (38.1%)	0.855

Antidiabetics	26 (41.3%)	25 (39.7%)	0.856
Statins	24 (38.1%)	14 (22.2%)	0.052
Antiplatelet	14 (22.2%)	8 (12.7%)	0.159
Warfarin	0 (0.0%)	1 (1.6%)	0.496†
Ischemic stroke subtype‡			0.962†
Large artery atherosclerosis	17 (27.0%)	19 (30.2%)	
Small vessel occlusion	36 (57.1%)	34 (54.0%)	
Cardioembolism	1 (1.6%)	2 (3.2%)	
Other determined etiology	1 (1.6%)	0 (3.2%)	
Undetermined etiology	8 (12.7%)	8 (12.7%)	
Premorbid mRS	1.2±1.6	1.3±1.8	0.677
Admission NIHSS	4.1±3.4	5.3±5.0	0.103
Discharge NIHSS	2.4±3.7	2.9±3.8	0.491
NIHSS (admission – discharge)	1.6±3.0	2.4±4.6	0.251

Discharge mRS	2.0±1.6	2.2±1.5	0.427
1 year mRS (60 & 62)	1.5±1.6	1.6±1.5	0.649

Values are number (percentage) or mean ± standard deviation.

*P for Student’s *t* test or Chi-square test.

†Fisher’s exact test.

‡TOAST classification

mRS and NIHSS denote modified Rankin scale and NIH stroke scale, respectively.
