# Supplementary material for: NINJ2 SNP may affect the onset age of first-ever ischemic stroke without increasing silent cerebrovascular lesions
Source: BMC Res Notes. 2012 Mar 20;5:155. doi: 10.1186/1756-0500-5-155 (PMC3368733; doi:10.1186/1756-0500-5-155)
Supplement: Additional file 7 — Table S6. Conditional logistic regression analysis to find independent factors associated with the rs11833579 A/A or G/A genotype vs. G/G genotype. [file 1756-0500-5-155-S7.PDF]

Supplementary Table 6 Conditional logistic regression analysis: factors associated with the rs11833579 A/A or G/A genotype vs. G/G genotype

Variable	Odds ratio (95% C.I.)	P
Hypertension	0.98 (0.53—1.81)	0.955
Diabetes mellitus	0.68 (0.53—1.52)	0.894
Dyslipidemia	0.77 (0.42—1.41)	0.397
Heart disease	0.71 (0.28—1.79)	0.703
NIHSS change (admission – discharge)	0.97 (0.90—1.05)	0.402
1 year mRS	0.96 (0.81—1.13)	0.615

NIHSS and mRS denote NIH stroke scale and modified Rankin scale, respectively.
