# Supplementary material for: NINJ2 SNP may affect the onset age of first-ever ischemic stroke without increasing silent cerebrovascular lesions
Source: BMC Res Notes. 2012 Mar 20;5:155. doi: 10.1186/1756-0500-5-155 (PMC3368733; doi:10.1186/1756-0500-5-155)
Supplement: Additional file 8 — Table S7. Multivariable analysis to predict admission NIH Stroke Scale (NIHSS) score. [file 1756-0500-5-155-S8.PDF]

**Supplemenatry Table 7 Multivariable Analysis to Predict Admission NIH Stroke Scale (NIHSS) Score**

| Variable | Odds ratio (95% C.I.) for admission NIHSS score | P |
|---|---|---|
| Rs11833579 genotype (GA or AA) | -0.12 (-1.31—1.07) | 0.843 |
| Statin medication | 0.49 (-0.94—1.92) | 0.499 |
| Premorbid mRS | 0.42 (0.04—0.80) | 0.032 |

C.I. denotes confidence interval.
